# AMPKα1 negatively regulates osteoclastogenesis and mitigates pathological bone loss

**DOI:** 10.1016/j.jbc.2023.105379

**Published:** 2023-10-21

**Authors:** Mariana S.P. Ribeiro, Lucas G.R. Venturini, Cesar A. Speck-Hernandez, Paulo V.G. Alabarse, Thais Xavier, Thaise M. Taira, Letícia F. Duffles, Fernando Q. Cunha, Sandra Y. Fukada

**Affiliations:** 1 Laboratory of Bone Biology, Department of BioMolecular Sciences, School of Pharmaceutical Sciences Ribeirão Preto, University of São Paulo, Ribeirão Preto, Brazil; 2 Department of Pharmacology, Ribeirão Preto Medical School, University of São Paulo, Ribeirão Preto, Brazil; 3 Center for Research in Inflammatory Diseases, CRID, Ribeirão Preto Medical School, University of São Paulo, Ribeirão Preto, Brazil

**Keywords:** AMPK, metabolism, osteoclast, osteoclastogenesis, osteoporosis

## Abstract

Osteoclasts are specialized cells responsible for bone resorption, a highly energy-demanding process. Focus on osteoclast metabolism could be a key for the treatment of osteolytic diseases including osteoporosis. In this context, AMP-activated protein kinase α1 (AMPKα1), an energy sensor highly expressed in osteoclasts, participates in the metabolic reconfiguration during osteoclast differentiation and activation. This study aimed to elucidate the role of AMPKα1 during osteoclastogenesis *in vitro* and its impact in bone loss in vivo. Using LysMcre/0AMPK⍺1f/f animals and LysMcre/0 as control, we evaluated how AMPKα1 interferes with osteoclastogenesis and bone resorption activity *in vitro*. We found that AMPKα1 is highly expressed in the early stages of osteoclastogenesis. Genetic deletion of AMPKα1 leads to increased gene expression of osteoclast differentiation and fusion markers. In addition, LysMcre/0AMPK⍺1f/f mice had an increased number and size of differentiated osteoclast. Accordingly, AMPKα1 negatively regulates bone resorption *in vitro*, as evidenced by the area of bone resorption in LysMcre/0AMPK⍺1f/f osteoclasts. Our data further demonstrated that AMPKα1 regulates mitochondrial fusion and fission markers upregulating Mfn2 and downregulating DRP1 (dynamics-related protein 1) and that Ctskcre/0AMPK⍺1f/f osteoclasts lead to an increase in the number of mitochondria in AMPK⍺1-deficient osteoclast. In our in vivo study, femurs from Ctskcre/0AMPK⍺1f/f animals exhibited bone loss associated with the increased number of osteoclasts, and there was no difference between Sham and ovariectomized group. Our data suggest that AMPKα1 acts as a negative regulator of osteoclastogenesis, and the depletion of AMPKα1 in osteoclast leads to a bone loss state similar to that observed after ovariectomy.

Osteoclasts are giant, multinucleated, and exclusive bone-resorbing cells in the human body with an important role in bone remodeling. The initial step of osteoclast differentiation is driven by the interaction of the cytokine receptor activator of NF-κB ligand (RANKL) with its receptor RANK (receptor activator of NF-κB) on myeloid progenitor cells that recruit tumor necrosis factor receptor–associated factor 6 (1). It then activates a cascade of downstream intracellular events, including the activation of transcription factors, NF-κB and nuclear factor of activated T cells, cytoplasmic 1 (NFATc1) (2).

To fulfill this function, during their differentiation and maturation, osteoclasts undergo a series of morphological and biochemical adjustments that require a high energy burden. The energy requirement is crucial not only during the fusion of multiple macrophages to form osteoclast but also to enable the adhesion and synthesis of osteolytic molecules necessary during bone resorption (3). Therefore, understanding how osteoclasts perform their function is also important to clarify how osteoclasts produce their energy (4).

In the context of energy production, the AMPK protein emerges as the main regulator of energy metabolism, since during metabolic stress it restores the energy supply to a physiological level. Its activation is triggered by increase in AMP and/or ADP concentrations or by high level of AMP–ATP ratio (5). Once activated, AMPK reduces cell metabolic expenses and stimulates the production of energy, inducing the mitochondrial biogenesis and fatty acid oxidation (6). AMPK is a protein expressed in all eukaryotes as a complex serine/threonine kinase heterotrimeric protein composed of three subunits: α that is a catalytic subunit and the β and γ subunits, both exhibiting regulatory roles.

Guo *et al.* (6) suggested that AMPK plays a regulatory role in osteoclast differentiation. They showed that AMPK activator metformin stimulates AMPK phosphorylation leading to an inhibition of NF-κB–extracellular signal–regulated kinase axis and reduce osteoclast number and function. Tong *et al.* (7) demonstrated that osteoprotegerin activates the AMPK and inhibits mammalian target of rapamycin–p70S6K axis, thereby inhibiting osteoclast differentiation while inducing autophagy. Previous findings indicated the presence of AMPKα1 and the absence of AMPKα2 in osteoclast population (8). Moreover, increased AMPKα1 expression is associated with osteoblast differentiation by the induction of autophagy (9). The present work provides evidences that AMPKα1 subunit acts as a negative regulator for osteoclast differentiation, and we suggest that this inhibitory effect is achieved through the modulation of energy production by the mitochondria.

## Results

### AMPKα1 negatively regulates osteoclastogenesis

**Figure 1:**
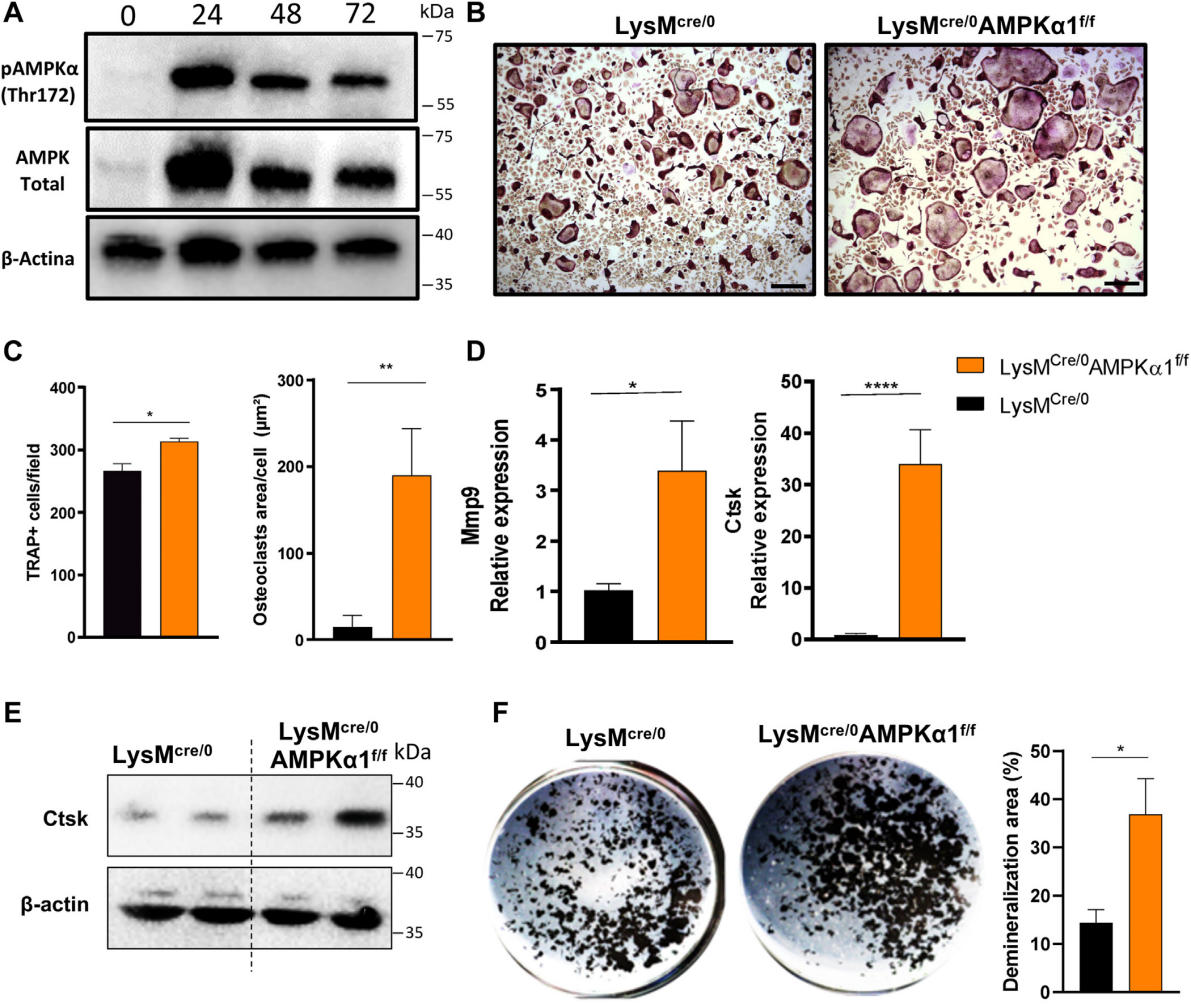
**AMPKα1 negatively regulates osteoclastogenesis.***A*, representative image of AMPKα1 and p-AMPKα1 protein expression during osteoclastogenesis, 0, 24, 48, and 72 h after RANKL stimulation. *B*, representative images of LysM^cre/0^ and LysM^cre/0^AMPKα1^f/f^ osteoclasts differentiation by TRAP staining. The scale bar corresponds to 200 μm. *C*, quantification of osteoclasts number and area *in vitro*. *D*, gene expression of osteoclast function markers (MMP-9 and cathepsin K). *E*, protein expression of cathepsin K. *F*, demineralization area of LysM^cre/0^AMPKα1^f/f^ and LysM^cre/0^ osteoclasts (n = 3, Student's *t* test ∗*p* < 0.05, ∗∗*p* < 0.01, ∗∗∗∗*p* < 0.0001). All experiments were performed three times. AMPKα1, AMP-activated protein kinase α1; MMP-9, matrix metallopeptidase 9; RANKL, receptor activator of nuclear factor-κB ligand; TRAP, tartrate-resistant acid phosphatase.

Bone marrow (BM) macrophages were stimulated with RANKL, and the expression of AMPKα1 was assessed over a 72 h of osteoclast differentiation. Our data show an upregulation of AMPKα1 expression after 24 h of RANKL stimulation, followed by a decrease after 48 and 72 h. Similarly, phosphorylation of AMPKα1 was upregulated at 24 h time point (Fig 1*A*). To study a possible role of AMPKα1 in osteoclast differentiation, we took advantage of a conditional *knockout* of AMPKα1 in macrophages (LysM^cre/0^AMPKα1^f/f^). We stimulated LysM^cre/0^AMPKα1^f/f^ macrophages for 72 h with RANKL, and osteoclasts were tartrate-resistant acid phosphatase (TRAP) stained and quantified. We observed an increase in the number and area of TRAP^+^ cells on LysM^cre/0^AMPKα1^f/f^ group when compared with LysM^cre/0^ control (Fig. 1, *B* and *C*). Subsequently, to analyze the impact of the AMPKα1 deletion in osteoclast function, we evaluated the gene expression of matrix metallopeptidase 9 (MMP-9) and cathepsin K. These molecules play a pivotal role in the degradation of the bone matrix, thereby contributing to osteoclast-driven resorption. LysM^cre/0^AMPKα1^f/f^ osteoclasts showed increased level of mRNA for MMP-9 and cathepsin K (Fig. 1*D*). The increased gene expression is reflected in the protein levels for cathepsin K, as demonstrated in Figure 1*E*. We also found an increased demineralized area induced by LysM^cre/0^AMPKα1^f/f^ osteoclasts when compared with the control (Fig. 1*F*). These results together demonstrate that AMPKα1 inhibits osteoclast formation and function *in vitro*.

### AMPKα1 deletion increases mitochondrial fusion and function in osteoclasts

**Figure 2:**
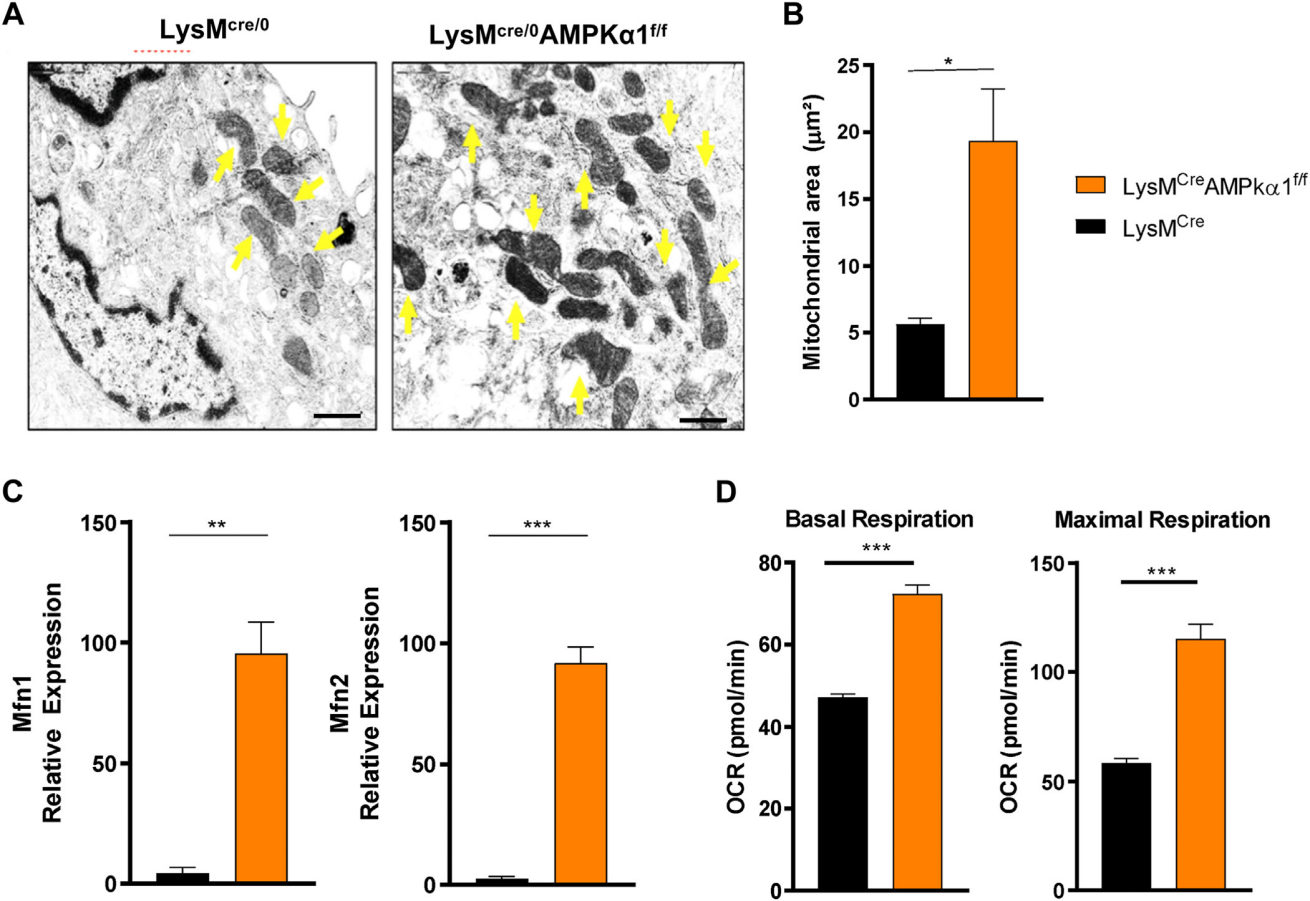
**AMPKα1 deletion increases mitochondrial fusion and function in osteoclasts.***A*, representative image of mitochondrias from LysM^cre/0^ and LysM^cre/0^AMPKα1^f/f^ differentiated osteoclasts. The scale bar corresponds to 1 μm. *B*, mitochondrial area in osteoclasts. *C*, mitofusin 1 and mitofusin 2 gene expressions. *D*, basal and maximal respiration from LysM^cre/0^AMPKα1^f/f^ and LysM^cre/0^ osteoclasts *in vitro* (n = 5, Student's *t* test ∗*p* < 0.05, ∗∗*p* < 0.01, ∗∗∗*p* < 0.001). All experiments were performed three times. AMPKα1, AMP-activated protein kinase α1.

Electron microscopy analysis of mitochondrial morphology shows that LysM^cre/0^AMPKα1^f/f^ osteoclasts exhibit increased number and size of mitochondria per osteoclasts (Fig. 2, *A* and *B*). It is known that mitochondria are not a static organelle and undergo continuous changes in morphology through processes of fission and fusion. This mechanism of mitochondrial biogenesis results in alterations in their number, distribution, and structural appearance. In order to investigate a potential link between AMPKα1 signaling and mitochondrial homeostasis, we extended the study assessing the expression of mitochondrial dynamic genes in osteoclasts and found that AMPKα1-deficient osteoclasts exhibit an increased expression of the two main genes involved in mitochondrial fusion mitofusin 1 (Mfn1) and mitofusin 2 (Mfn2) when compared with the control (Fig. 2*C*). Since AMPK acts as a metabolic sensor governing mitochondrial function, our next question aimed to assess whether the consequences of AMPK deletion on bone loss might correlate with potential alterations in the metabolic activity of bone-resorbing osteoclasts. Our data show that LysM^cre/0^AMPKα1^f/f^ osteoclasts exhibit an increased oxidative phosphorylation energy production evidenced by increased basal and maximal respiration (Fig. 2*D*).

### AMPKα1 deletion inhibits mitochondrial fission

**Figure 3:**
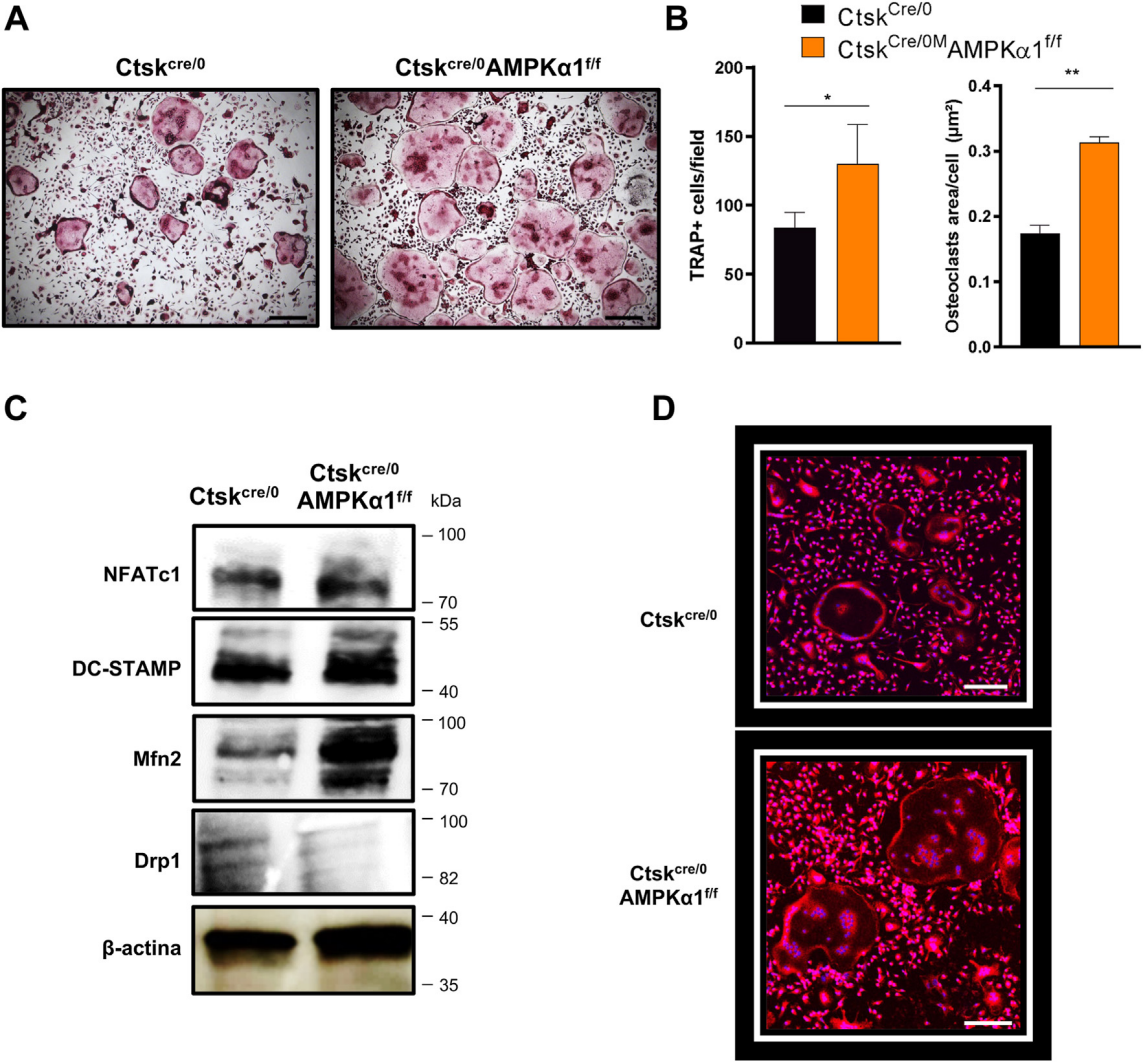
**AMPKα1 deletion inhibits mitochondrial fission.***A*, representative images of Ctsk^cre/0^ and Ctsk^cre/0^AMPKα1^f/f^ osteoclast differentiation by TRAP staining. The scale bar corresponds to 200 μm. *B*, quantification of osteoclast number and area *in vitro*. *C*, protein expression of osteoclasts and mitochondrial dynamics markers: NFATc1, DC-STAMP, mitofusin 2, Drp-1, and β-actin as positive control. *D*, representative image of Ctsk^cre/0^ and Ctsk^cre/0^AMPKα1^f/f^ osteoclasts labeled with MitoTracker Red. The scale bar corresponds to 200 μm. (n = 5, Student's *t* test ∗*p* < 0.05 and ∗∗*p* < 0.01). All experiments were performed two times. AMPKα1, AMP-activated protein kinase α1; Drp1, dynamics-related protein 1; TRAP, tartrate-resistant acid phosphatase.

To elucidate the role of AMPKα1 modulating the dynamic processes of mitochondrial fusion and fission, we took advantage of a conditional *knockout* of AMPKα1 in osteoclasts (CtsK^cre/0^AMPKα1^f/f^). Our findings indicate that CtsK^cre/0^AMPKα1^f/f^ also exhibited increased osteoclast formation (Fig. 3, *A* and *B*). We also evaluated whether AMPKα1 deficiency in osteoclasts would impact markers associated with mitochondria fusion and fission. Our data show that AMPKα1-deficient osteoclasts exhibit an increase in osteoclast markers (NFATc1 and DC-STAMP) along with an increase in the fusion marker Mfn2. Conversely, the fission marker dynamics-related protein 1 (Drp1) displays a reduction in the expression level (Fig. 3*C*). These observations are further validated by the mitochondrial abundance assessed using MitoTracker, revealing an overall augmentation in mitochondrial content (Fig. 3*D*). These data together suggest that AMPKα1 might regulate mitochondrial dynamics within osteoclasts.

### Deletion of AMPKα1 increases osteoclast differentiation and function *in vivo*

**Figure 4:**
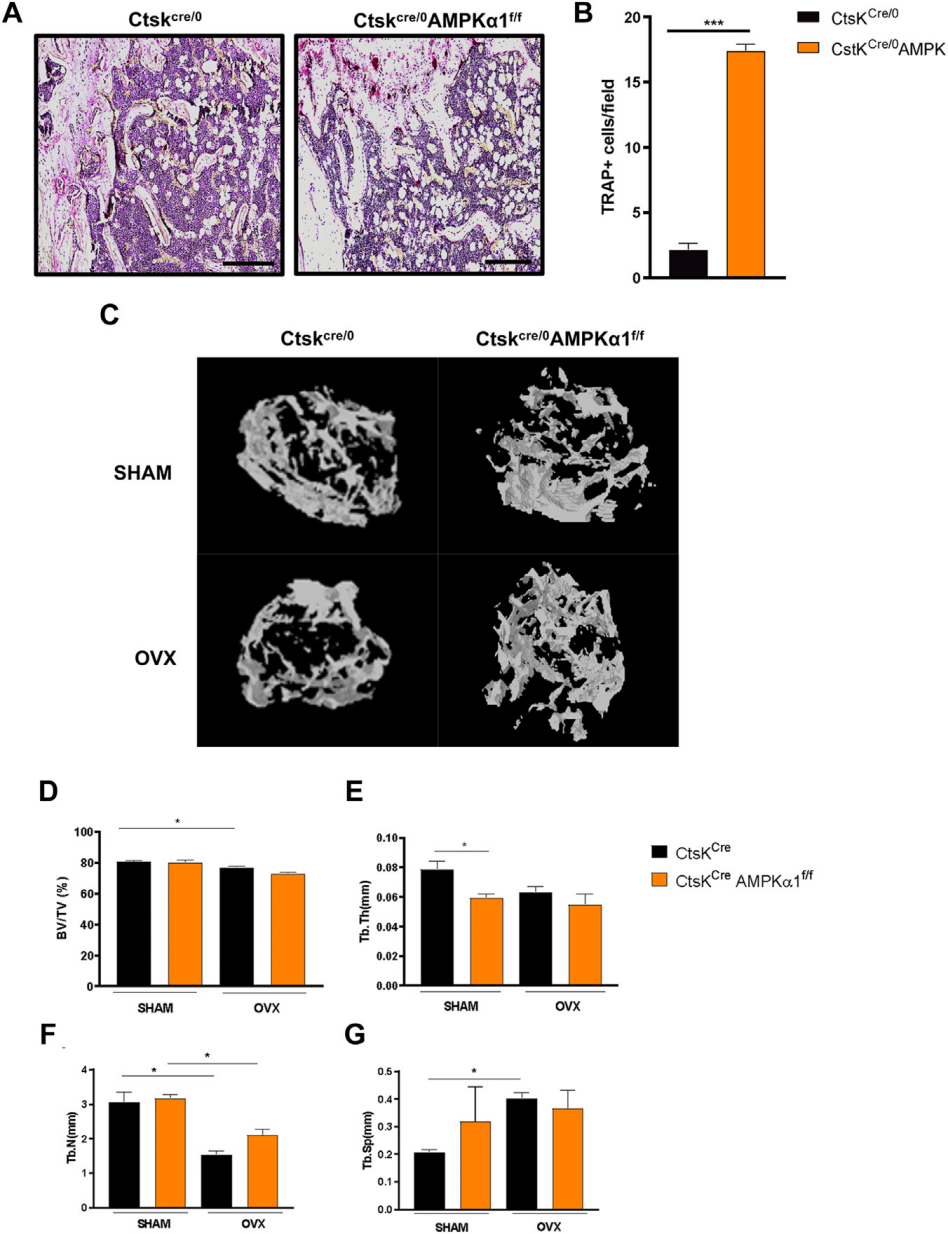
**AMPKα1 deletion leads to increase in osteoclast’s differentiation and function *in vivo*.***A*, representative image of TRAP^+^ cells in a femur section of Ctsk^cre/0^ and Ctsk^cre/0^AMPKα1^f/f^ mice. The scale bar corresponds to 200 μm. *B*, TRAP+ cells. *C*, representative image of a computed microtomography of Ctsk^cre/0^ and Ctsk^cre/0^AMPKα1^f/f^ mice under or not under ovariectomy. Trabecular parameters: (*D*) bone volume/tissue volume, (*E*) trabecular thickness, (*F*) trabecular number, (*G*) trabecular separation (n = 3, Student's *t* test and ANOVA following by Tukey for multiple comparisons ∗*p* < 0.05, ∗∗∗*p* < 0.001). All experiments were performed three times. AMPKα1, AMP-activated protein kinase α1; TRAP, tartrate-resistant acid phosphatase.

To assess the role of AMPKα1 in osteoclasts *in vivo* and its biological effect, we generated mice with a conditional deletion of AMPKα1 in osteoclasts (Ctsk^cre/0^AMPKα1^f/f^). Histological analysis of distal femur revealed increased number of osteoclasts (TRAP^+^ cells) in the Ctsk^cre/0^AMPKα1^f/f^ mice when compared with the control (Fig. 4, *A* and *B*). Next, we evaluated the impact of the deletion of AMPKα1 on a well-established mouse model of bone loss induced by ovariectomy (Fig. 4*C*). Microcomputerized tomography analysis of the trabecular microstructure showed that bone volume/total volume (Fig. 4*D*), trabecular number (Fig. 4*E*), and trabecular seperation (Fig. 4*G*) displayed changes after ovariectomy both in Ctsk^cre/0^AMPKα1^f/f^ and Ctsk^cre/0^ confirming the success of the model. And when analyzed the trabecular thickness (Fig. 4*E*) in sham mice, the AMPKα1 deletion itself leads to a decrease in trabecular thickness, suggesting that the absence of AMPKα1 selectively in osteoclasts could lead to bone loss in sham mice. These data indicate that AMPKα1 in osteoclasts exhibits a protective role for the bone microarchitecture integrity.

## Discussion

Cell differentiation and bone resorption demand a high amount of energy, and AMPK plays a crucial role regulating cellular energy supply. This protein is expressed in all eukaryotic cells and highly expressed in both osteoclasts and osteoblasts (10). Previous studies by Lee *et al.* (11) demonstrated that RANKL increases expression of AMPK⍺1 during osteoclast differentiation process. In the present study, we confirm earlier findings and show that RANKL potentiates protein expression of AMPK⍺1 and its phosphorylated form (pAMPK⍺1) within the first 24 h of differentiation.

To investigate the role of early phosphorylation of AMPK on osteoclastogenesis, we generated mice with conditional deletion of AMPK⍺1 in myeloid lineage cells, which serve as osteoclast precursors, using LysM^cre/0^AMPKα1^f/f^ cells. Our findings show that the deletion of AMPKα1 in progenitor cells leads to increased number and area of osteoclasts and consequently increased demineralization area by osteoclasts. Osteoclast activity markers essential for bone matrix degradation. MMP-9 and cathepsin K, (12, 13) were genetically upregulated in osteoclasts lacking AMPKα1. Altogether, these results suggest that AMPKα1 plays a negative regulatory role in osteoclast differentiation.

AMPK, as an energy sensor, is known to regulate mitochondrial dynamics in various cell types (14). Galic *et al.* (15) demonstrated that AMPKα1 deletion suppresses the expression of mitochondrial enzymes in macrophages. Under energy deprivation conditions, AMPK regulates mitochondrial fission and mitophagy (16). Mitochondria are in a constant balance of division and fusion, which is essential for maintaining their quality and quantity. While Mfn1 and Mfn2 are essential for mitochondrial fusion (17), Drp1 is important for mitochondrial fission (18). A recent study demonstrated that under energy stress conditions, AMPK translocates from the cytosol to the mitochondria-associated endoplasmic reticulum membrane and directly interacts with Mfn2 to regulate autophagy in myofibroblasts (19). However, the precise involvement of AMPK⍺1 on mitochondrial dynamics during osteoclastogenesis is not clear. In the present study, the increased gene expression of Mfn1 and Mfn2, along with the larger mitochondrial area observed in osteoclasts lacking AMPK⍺1, suggests that AMPK⍺1 regulates mitochondrial biogenesis and fusion, while promoting mitochondrial fission through the upregulation of Drp1. Osteoclasts is a product of cell fusion, thus it is expected to have an increased number of mitochondria, especially because their function requires a high energy load. It is conceivable that the reduced activation of AMPK observed at late stage of osteoclast is a mechanism to enhance energy supply by increasing mitochondrial number. In our study, the deletion of AMPK⍺1, a well-known regulator of energy production, results in increased oxidative phosphorylation in osteoclasts. Lemma *et al.* (3) reported a similar increase in oxidative phosphorylation during osteoclast differentiation, suggesting a critical role for AMPK⍺1 in regulating osteoclast differentiation through enhanced oxidative phosphorylation energy production.

While previous studies, using nonselective AMPK inhibitors or total AMPK⍺1 deletion, have shown a reduction in bone parameters, our study is the first to elucidate the specific role of AMPK⍺1 in osteoclast on bone metabolism. In agreement with previous studies, our data demonstrate a reduction in the bone parameters including bone volume/total volume, trabecular thickness, and trabecular number in animals with AMPK⍺1 deletion specifically in osteoclasts. By selectively deleting AMPK⍺1 only in osteoclasts, we are able to dissect the role of the ⍺1 subunit, excluding the influence of other subunits and AMPK in other cell type involved in bone remodeling such as in osteoblasts. Bone loss studies using general modulators or animals with total AMPK⍺1 deletion do not allow to discern whether the observed effect is due to AMPK⍺1 deficiency in osteoclasts, osteoblasts, or even in another cell type. Our histological data show that selective deletion of AMPK⍺1 in osteoclasts increases the number of TRAP-positive cells. This suggests that the selective deletion of AMPK⍺1 in osteoclast leads to a higher susceptibility to osteoporosis as a consequence of increase in osteoclasts. Both our *in vitro* and *in vivo* findings confirm that AMPK⍺1 in osteoclasts plays a central role preventing bone loss. In conclusion, these data strongly indicate that AMPKα1 controls osteoclast differentiation and function by the regulation of mitochondrial dynamics and energy production.

## Experimental procedures

### Animals

In this study, mice with C57BL/6 background were used. Male animals aged 6 to 8 weeks, LysM^cre/0^, LysM^cre/0^AMPK⍺1^f/f^, and females aged 12 to 16 weeks, Ctsk^cre/0^ and Ctsk^cre/0^AMPK1^f/f^, were used. The animals were kept in the specific vivarium during the experiment, with a 12 h light–dark cycle, controlled temperature, and humidity. Water and food were administered ad libitum. The study was approved by the Research Ethics Committee of the University of São Paulo.

### Osteoclast cell culture

The differentiation of murine osteoclasts was performed from primary culture using BM cells. For this, femur and tibia of LysM^cre/0^ and LysM^cre/0^AMPK⍺1^f/f^ mice were aseptically removed and BM fluxed. The cells were cultured for 3 days in α-MEM culture medium (GIBCO, Invitrogen) supplemented with 10% fetal bovine serum, 100 units/ml penicillin, and 100 mg/ml streptomycin (ThermoScientific) in the presence of 30 ng/ml macrophage colony-stimulating factor (M-CSF; R&D Systems). Nonadhered cells were removed and discarded, and attached cells (preosteoclasts) were cultured in osteoclastogenic medium with 30 ng/ml M-CSF and 10 ng/ml RANKL (R&D Systems).

### Osteoclast number by TRAP staining

The detection of osteoclasts was performed by the reaction of TRAPs (KIT SIGMA 387-A). The cells were first fixed with citrate buffer for 20 min, washed with PBS, and incubated at 37 °C in the dye solution containing tartrates for 40 min, protected from light, according to the manufacturer's instructions. Subsequently, cells stained in purple containing three or more nuclei (TRAP+ cells) were considered osteoclasts. TRAP+ cell number and size were quantified using the imaging program ImageJ software (National Institutes of Health).

### Computed microtomography

Samples from the left femurs of Ctsk^cre/0^ and Ctsk^cre/0^AMPK1^f/f^ animals were dissected and fixed in 10% formaldehyde to standardize their positioning during microCT. Measurements of bone patterns were performed using the Data Viewer and CTAn (Bruker microCT) imaging programs. The images of the femurs, obtained by microCT, were adjusted to measure the trabecular bone, excluding the cortical part (1 cm above grow plate), and a total of 1 cm was used for the analysis.

### Transmission electron microscopy

To count the mitochondrial number and area, a primary culture of BM cells from Ctsk^cre/0^ and Ctsk^cre/0^AMPK1^f/f^ mice was performed, and after 3 days of stimulation with RANKL, the culture was fixed for 2 h in glutaraldehyde (2% in phosphate buffer) and included in Embed. The ultrafines were prepared and viewed using a transmission electron microscope (Jeol JEM-100 CXII equipped with a Hamamatsu ORCA-HR digital camera). Photos of 10 osteoclasts were randomly taken from the same slice, and the number and mitochondrial area of these osteoclasts were measured using ImageJ software.

### Ovariectomy model

The Ctsk^cre/0^ and Ctsk^cre/0^AMPK1^f/f^ mice were anesthetized, and an incision was made on each flank (right and left). After the identification of the ovaries, their peduncles were ligated, and then the ovarian tissues were resected. As a control, mice were similarly manipulated, without resection of the ovarian tissue (Sham/OVX group). After a period of 30 days, the animals were euthanized and femur were collected.

### Quantitative PCR

For the quantitative PCR assay, 200,000 preosteoclasts were plated in 24 wells in osteoclastogenic medium. Samples were collected at time 0, 24, 48, and 72 h. Total RNA isolation was performed using the Promega SV Total Isolation System Kit (Promega). Then, 500 ng of mRNA was used for the complementary DNA synthesis using reverse transcriptase reaction kit (High capacity cDNA Reverse Transcription kit from Applied Biosystems; catalog no.: 4368813). The expression of genes for *Praak1* (AMPKα1), *Ctsk* (cathepsin K), and *Mmp9* (metalloproteinase 9) was analyzed by TaqMan, and *Mfn1* and *Mfn2*, were analyzed by Sybr Green (Applied Biosystems).

### Western blotting

For Western blot assay, 200,000 preosteoclasts were plated in 24-well plate in osteoclastogenic medium for 3 days. The protein extract (10 μg for DC-STAMP, 4 μg for cathepsin K, and 40 μg for AMPKα1) was evaluated using a nitrocellulose membrane and the primary antibodies anti-AMPKα1 (ab3759) and anti-drp1 (ab8570) (Cell Signaling), anti-pAMPKα1 (PA517831) (Thermo Fisher), anti-cathepsin K (ab66237), anti-NFAT2 (ab2796), anti-Mfn2 (ab56889), antibeta actin (ab8226) (Abcam), and anti-DC-STAMP (clone 1A2, catalog no.: MABF39-I) (Merck KgaA) and the secondary antibodies antimouse (ab6789, Abcam) and anti-rabbit (ab6728, Abcam).

### MitoTracker Red assay

Cells were cultured on coverslips in 24-well plates for 4 days at 37 °C with 5% CO_2_ in a medium containing 10 ng/ml RANKL and 10 ng/ml M-CSF. Following the incubation period, cells were fixed with 4% paraformaldehyde for 10 min and treated with 1 μM MitoTracker Red CMROX (Invitrogen; catalog no.: M7512) for 30 min at 37 °C. Cells were mounted using Fluoroshield with 4′,6-diamidino-2-phenylindole (Sigma–Aldrich; catalog no.: F6057) and observed using a Leica SP5 Confocal Microscope. Image analysis was performed using the ImageJ software program.

### Statistical analysis

Data are presented as mean ± SD from three independent experiments. Statistical comparisons between two groups were determined by *t* test using GraphPad Prism 8.0.1 statistical software (GraphPad Software, Inc). *p* < 0.05 was considered as statistically significant.

## Data availability

All the data described in this study are contained within the article.

## Conflict of interest

The authors declare that they have no conflicts of interest with the contents of this article.
